# Methods for multi-omic data integration in cancer research

**DOI:** 10.3389/fgene.2024.1425456

**Published:** 2024-09-19

**Authors:** Enrique Hernández-Lemus, Soledad Ochoa

**Affiliations:** ^1^ Computational Genomics Division, National Institute of Genomic Medicine, Mexico City, Mexico; ^2^ Center for Complexity Sciences, Universidad Nacional Autónoma de México, Mexico City, Mexico; ^3^ Department of Obstetrics and Gynecology, Cedars-Sinai Medical Center, Los Angeles, CA, United States

**Keywords:** multi-omics, data integration, statistical and probabilistic modelling, regulatory models, LASSO, cancer biology

## Abstract

Multi-omics data integration is a term that refers to the process of combining and analyzing data from different omic experimental sources, such as genomics, transcriptomics, methylation assays, and microRNA sequencing, among others. Such data integration approaches have the potential to provide a more comprehensive functional understanding of biological systems and has numerous applications in areas such as disease diagnosis, prognosis and therapy. However, quantitative integration of multi-omic data is a complex task that requires the use of highly specialized methods and approaches. Here, we discuss a number of data integration methods that have been developed with multi-omics data in view, including statistical methods, machine learning approaches, and network-based approaches. We also discuss the challenges and limitations of such methods and provide examples of their applications in the literature. Overall, this review aims to provide an overview of the current state of the field and highlight potential directions for future research.

## 1 Multi-omic integration

Multi-omics integration is a series of methods and techniques aimed at the joint interpretation of different omics which has been made possible by the accumulation of measurements obtained with different high-throughput technologies and their deposit in databases – especially public ones –. In the most ambitious studies, reserachers even talk of pan-omics, the simultaneous measuring of a biological systems with all the omic technologies available. Current multi-omic approaches, often involve large amounts of measurements, with different *units* and *dynamic ranges*, and not necessarily synchronous. Hence, multi-omics constitute a complex perspective that demands its own statistical tools. It is thus an approach that has had a long *gestation* period, during which it was necessary to standardize high-performance technologies, achieve a minimum number of samples required to meet statistical requirements, and adjust computational tools to new objectives. This article will address the computational tools that have been developed, as they constitute both precedents and methods of interest. Although the main focus will be on applications of multi-omic integration to build semi-mechanistic models of regulatory programs in cancer, most of what we will discuss is indeed applicable to other instances such as the study of other chronic and infectious diseases, and even in agricultural or ecological studies.

Multi-omic integration is thus a relevant topic that has been generating a lot of interest in recent times. One comprehensive example of how the ideas behind systematic integration approaches to this problem have been established can be found in the work by Wu and collaborators Wu et al. (2019) which discusses the integration of multi-level omics data through the use of variable selection methods. The focus is on addressing the challenges and methodologies associated with integrating diverse types of omics data, such as genomics, transcriptomics, proteomics, and metabolomics, to provide comprehensive insights into biological systems. This comprehensive review highlights the importance of integrating multi-omics data to understand complex biological phenomena and improve the precision of disease diagnosis and treatment. It outlines several approaches for data integration, emphasizing the significance of variable selection techniques in handling high-dimensional data and identifying relevant biomarkers.

The authors also discuss different variable selection methods, including LASSO (Least Absolute Shrinkage and Selection Operator), elastic net, and other regularization techniques, that are commonly used to manage the complexity of multi-omics data. These methods help in reducing the dimensionality of the data by selecting the most informative variables while discarding the less relevant ones. They also addresses the limitations of current variable selection methods, such as their sensitivity to data heterogeneity and potential for producing false findings. It suggests that integrating robust statistical techniques and leveraging network-based approaches can improve the accuracy and reliability of the findings thus providing a comprehensive overview of the current state of multi-level omics data integration using variable selection methods and offers insights into future research directions to enhance the integration process. This work is indeed an authoritative source on the issues discussed, strongly founded on statistical thinking with a view on clinical applications.

### 1.1 Generalities of computational integration

The promise of multi-omics integration is hence to provide a more *complete* perspective of complex biosystems such as cancer by considering different functional levels, rather than focusing on a single aspect of this heterogeneous phenomenon. Specifically, three objectives have been mentioned:

• discover molecular mechanisms, as well as their association with phenotypes;

• group samples or improve the characterization of known groups, and;

• predict phenotypes (Kristensen et al., 2014; Bersanelli et al., 2016a).

The last two objectives can be ordered in a successive manner, first we find samples that are grouped, then we predict what will happen with the new samples that are integrated into the groups. A byproduct of such a succession would be the identification of biomarkers, which allow the recognition of a sample’s belonging to a group. These two objectives also match known statistical learning problems, such as *clustering*, classification, and regression. On the other hand, the discovery of molecular mechanisms, which ideally would follow the identification of markers, relies on network inference, and largely requires the generation of validation data.

**FIGURE 1:**
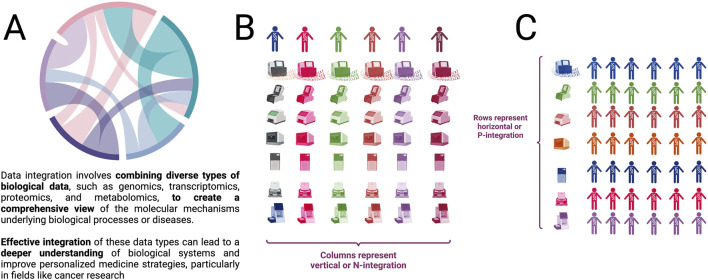
**(A)** Overview of data integration. **(B)** Vertical or N (also called subject-based) integration is represented by columns in which every subject (sample, patient, etc.) has integrated information for different omic technologies. **(C)** Horizontal or P (also called omic-based) integration is represented by columns in which every omic technology (DNA sequencing, RNASeq, Methylation, ATAC-Seq, Hi-C-seq, etc.) is measured in many (ideally all) subjects. (Figure created with BioRender.com).

Using different names, multi-omics integration has been divided according to the moment of integration and the object to be integrated (see Figure 1A). It is called vertical integration or *N-integration* (Figure 1B) when different omics are incorporated referred to the same samples, that is, the use of concurrent observations of different functional levels. This is the type of integration that this work aspires to. On the other hand, horizontal integration or *P-integration* (Figure 1C) adds studies of the same molecular level, made on different subjects, to increase the sample size (Ulfenborg, 2019; Rohart et al., 2017).

Additionally, *early* and *late* integration are discussed according to the time of execution. Early integration refers to the concatenation of measurements obtained with different omics from the beginning, before any classification or regression analysis, which disregards heterogeneity between platforms. On the other hand, late integration combines multiple predictive models, obtained separately for each omics, ignoring interactions between levels and the possibility of synergy or antagonism (Rohart et al., 2017); this is the first type of multi-omics integration that occurred and despite delivering extremely useful results, it has progressively been abandoned for other approaches. Although less discussed, an intermediate approach has also been proposed, in which a single set of data is modeled after transforming the omics through separate analysis, which respects the diversity of platforms, without necessarily capturing interactions between functional levels (Kim et al., 2014).

In addition to the compatibility problem between platforms, multi-omics integration faces challenges in terms of noise, which increases with the number of variables contributed by each omics; to dimensionality, since the number of variables always exceeds the sample size, and to the interpretability of the final model, which becomes more difficult as more variables are added (Kristensen et al., 2014; Tini, 2017). To resolve the compatibility issue, different normalizations are used, after independent pre-processing and according to the requirements of each platform. The normalization method required by most tools is the *standardization* of the concatenated data, that is, bringing all values to a mean of zero and variance of one, regardless of the omics of origin. When the number of variables and noise differs between platforms, the normalization of matrix factorization analysis (MFA) is recommended, which divides the data block of each omics by the square root of the first eigenvalue, in this way, all platforms have the same weight in the analysis. To prevent the largest block from dominating the analysis, it has also been used to divide each block by the square root of the number of variables or the total variance (Garali et al., 2018; Lock et al., 2013) and an algorithm has even been proposed to detect the optimal normalization method (Ciucci et al., 2017).

The problems of dimensionality and interpretability can be faced at the same time with the application of concise multivariate methods. Multivariate methods simultaneously study multiple variables, being part of early integration. To make them concise, a penalty is added to the fitting function, which contracts the coefficients so that some variables end up with a zero coefficient and leave the model, improving its interpretability, while at the same time allowing adjustment despite excess dimensions. These methods also use the decomposition of data matrices, in particular singular value decomposition (James et al., 2013), producing well-founded, fast statistical tools, and, as described below, ready for application to questions in complex phenotypes such as cancer.

In turn, network construction displays interactions between pairs of entities, normally without restriction as to their origin, allowing the integration of any set of omics. The focus on a subset of interpretable interactions is made, either by using a priori known functional networks such as metabolic or signaling pathways or by using significance or connectivity thresholds. In this case, the problem of excess variables with respect to the sample size largely depends on the inference method, although tools have been proposed to identify the optimal sample size according to the acceptable classification error (Tarazona et al., 2020).

## 2 Data integration classification

**FIGURE 2:**
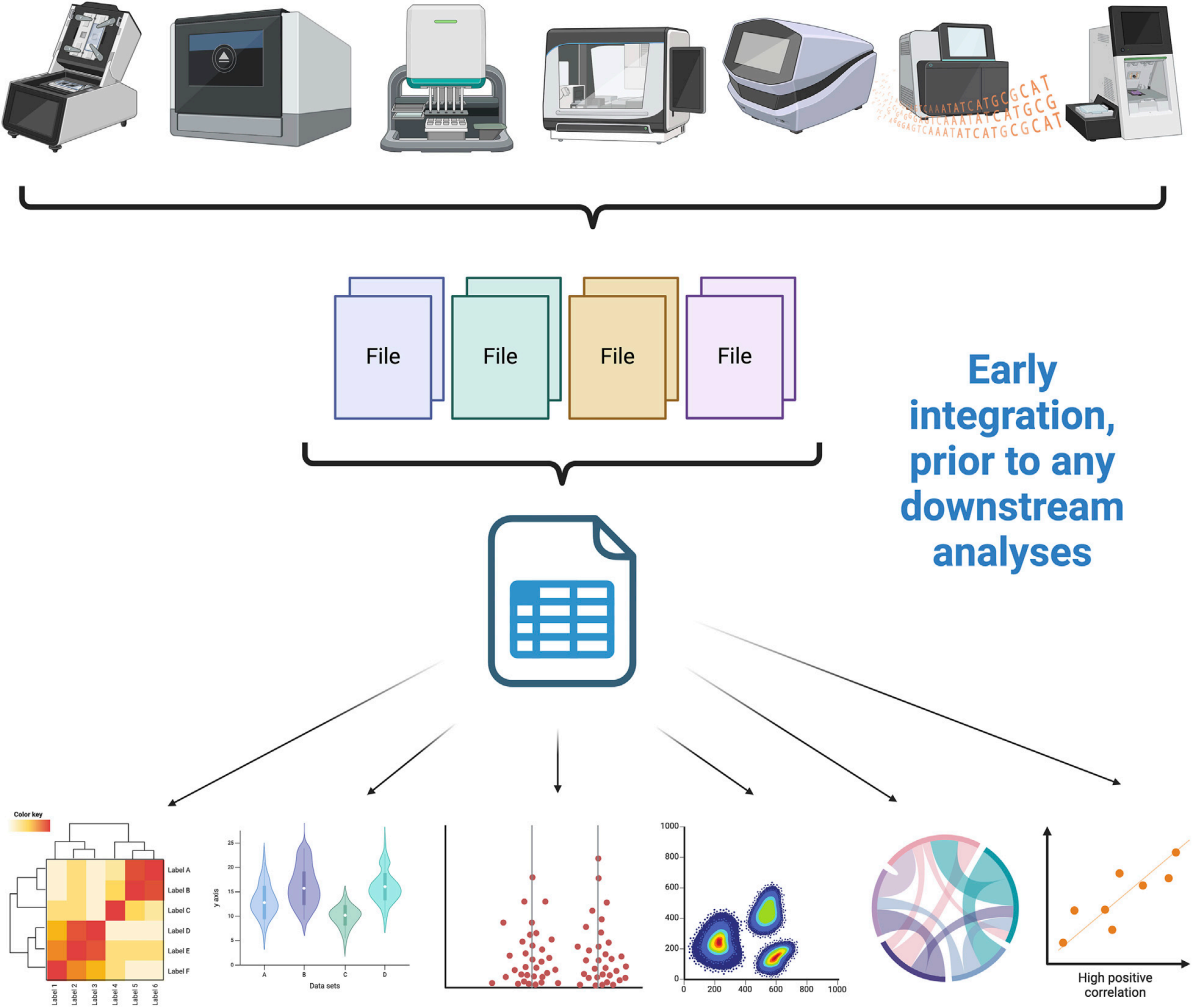
Early integration is carried out by concatenating the different omic datasets prior to any data analysis. (Figure created with BioRender.com).

**FIGURE 3:**
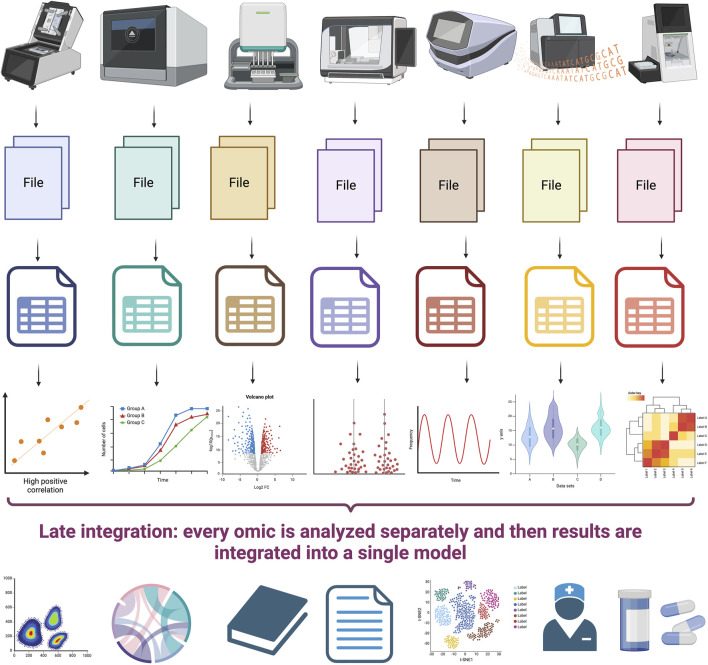
Late integration is carried out by analyzing each omic datasets separately to later integrate the different results. (Figure created with BioRender.com).

Data integration methods are the technical means by which data from different sources is combined into a single unified dataset, often for analytical reasons. There are several different classes of data integration methods that can be used to facilitate data integration, including extract, transform, and load (ETL) processes, data virtualization, data federation, and other methods. Each of these methods offers distinct advantages and disadvantages in terms of cost, scalability, and speed, so it is important to understand the differences between them in order to select the best method for a given data integration project. Particularly relevant to the discussion on the integration of multi-omics is the fact that multi-omic data often comes from high-throughput experimental sources with different –often disparate– assumptions, dynamic ranges and noise levels. Also, statistical approaches to integration must take into account –albeit often in an incomplete way– the biology behind the different omic sources and their relationships and the fact that, for obvious reasons, the different features are often interdendent. In what follows, we will discuss the different classes of data integration methods and their relative strengths and weaknesses (see Figures 2, 3).

### 2.1 Late integration

The *cluster-of-clusters* (CoCA) analysis is perhaps the late integration method that has had the most impact, being the base tool of *The Cancer Genome Atlas* (TCGA), one of the largest collection of standardized multi-omics data in contemprary biomedicine (Network et al., 2012). It is a consensus clustering algorithm based on the groups identified separately in each omics. Although it was introduced for breast cancer data (Network et al., 2012), later on, a decision tree was chosen to group, for instance, gynecological tumors (Berger et al., 2018), as CoCA grouped them by type of cancer. Although it disregards the relationship between omics, this tool identified patterns that allow for speculation about the effect one omics has on the other. For example, it was in Berger et al. (2018) that the coincidence between the basal subtype, the highest DNA hypomethylation and high genomic instability was described, which could very well be the result of altered transposon methylation, but proving it requires more data.

Leaving aside clustering algorithms, ActivePathways adds gene significance from different omics analyses and performs a functional enrichment analysis, both for the integrated list and for separate significances, thus determining which enrichment depends on which evidence. When applied to copy number variants (CNVs) and gene expression data from the *METABRIC* collaboration (Curtis et al., 2012), Paczkowska and coworkers were able to identify pathways whose enrichment depends on data integration, such as the negative regulation of apoptotic processes in the enriched Her2 subtype, and suggest markers (Paczkowska et al., 2020).

As it can be seen, late integration tools take independent results and do not consider relationships between omics, without this in any way preventing the satisfaction of classification or functional enrichment goals (for use cases, pros and cons of these methods see Supplementary Table S1). Strictly speaking, the only goal of multi-omic integration that demands early integration is that of discovering multi-omic molecular mechanisms. Even so, there are examples of early integration for all kinds of purposes.

### 2.2 Early integration

In contrast to adding independent results, concatenating the data from the beginning allows for observing joint effects. However, concatenation also produces very large data matrices, which generally require dimensionality reduction strategies for their analysis. Dimensionality reduction consists of constructing a reduced set, q , of new variables, through linear combination of the original variables, p . The new variables are called *principal axes*, *eigenvectors* or variables, components or *latent factors* and are formed by p coefficients (loadings), with at least one value different from zero. There is no standard way of choosing the size of q , but it is usually chosen according to the point where the variance explained by each latent factor stabilizes. To find these latent components, we seek to maximize the variance that each one represents, but maintaining orthogonality between them, that is, capturing the greatest amount of different information (Meng et al., 2016).

The most common dimensionality reduction strategy is *principal component analysis* (PCA), to represent complex data in the plane. Although there are as many flavors as data qualities, such as *correspondence analysis*, *non-negative matrix factorization* (NMF), *canonical correlation analysis* (CCA), *partial least squares regression* (PLS), or *co-inertia analysis* (CIA) (Meng et al., 2016). *Multiple factor analysis* (MFA) was built on PCA, especially useful for multi-omic integration, by considering the structure of the data, normalizing each block of variables (omics) with its first eigenvalue and then obtaining the principal components of the complete concatenated matrix. MFA normalization tries to give all omics a weight in the results relative to their variance - and not their size - and solves the problem of early integration with heterogeneity between platforms (De Tayrac et al., 2009).

*Multi-omics Gene Set Analysis*, MOGSA is a functional enrichment tool specifically designed for single cell data, but which exemplifies very well what is pursued with early integration. To estimate enrichment, MOGSA projects target sets onto the MFA axes, generating an enrichment value for each one. A high enrichment value implies variables that explain a large proportion of global information, from one or several blocks of data, but always discarding exclusive effects of one block, such as the batch effect associated with a platform. In this way it is possible to find functional enrichment over disparate omics, without mapping the different variables to the same genes, as would be necessary for the aforementioned ActivePathways. Nor is it necessary to have significance values per omics, eliminating the comparison between groups that would require a differential expression analysis. In this case, only the membership of each variable, of each block, in the target sets is needed. In addition, the decomposition allows excluding factors that are not of interest, improving interpretability (Meng et al., 2019).

Another tool that starts from PCA is *Joint and Individual Variation Explained* (JIVE), which decomposes the concatenated matrix into submatrices - of lower rank - of shared variance, individual variance, and noise. In addition to reducing dimensionality, JIVE allows for the visual exploration of the shared variance matrix and from that, the identification of potential biomarkers (O’Connell and Lock, 2016). When analyzing miRNA and transcript expression data from glioblastoma, JIVE finds more individual variance than shared variance between the omics, and more in transcript data than in miRNA data. In turn, the shared structure recovers more variance from miRNAs than from transcripts. Since the structures are orthogonal, the information in the shared variance matrix, where cancer subtypes can be appreciated, is not related to the individual matrices. Since originally the subtypes were identified by grouping gene expression, the weight of miRNAs was an unexpected result. By examining the coefficients on the shared structure, the authors were able to identify both transcripts and miRNAs with roles in the disease (Lock et al., 2013).

In addition to functional enrichment and the search for shared structures, matrix decomposition can be used to group tumors, as Cantini and collaborators did with different tools, with different assumptions. Working with simulated data, intNMF and iCluster identified the best groupings, with iCluster being worse, but allowing all types of data and distributions, without the restriction of negative values of NMF, and finding a common latent component between omics (Shen et al., 2009). Working with data from TCGA, MCIA, *generalized canonical correlation analysis* RGCCA and JIVE are the best for finding factors associated with clinical characteristics or biological functions. The RGCCA, which considers different and non-shared latent factors per omics, performs best on breast cancer data. It is suggested that this identification of latent factors per omics not only allows finding shared biological processes, but also processes that are complementary between data blocks (Cantini et al., 2021).

RGCCA has also been used to identify metabolites with potential as markers for hepatic cancer or cirrhotic tissues. *Multi-Omic inTegrative Analysis* (MOTA) is a tool that uses RGCCA to efficiently estimate the correlation between elements of different omics. On the premise that phenotypes not only diverge in the abundance of molecules, but also in the way they are connected, MOTA estimates differential correlations with RGCCA results and only connects pairs of elements with values above a pre-specified threshold. In this way, MOTA produces a lean network and a score per node. This score reflects both the connectivity of the node and its differential expression, and facilitates the selection of nodes of interest among those with the highest score. In their comparison of hepatic tumors and cirrhotic tissues, the authors find that the top 30 transcripts with the best score are enriched in cancer genes and processes related to hepatic cancer, which does not happen with other equivalent tools (Fan et al., 2020). Thus, the identification of possible markers is added to the list of objectives achieved with early integration techniques.

RGCCA is generalized by the ability to analyze more than two blocks of variables simultaneously and is regularized by including a penalty on the coefficients (Tenenhaus et al., 2014). This is not a lean method because the penalty it uses, known as *ridge*, does not set any coefficient to zero, but simply brings it closer to this value. This penalty reduces the variance of the fit and produces coefficients whose absolute value is used to discern the most relevant variables in the components. However, the cut-off point on *the most relevant* is always arbitrary. Instead, LASSO and ENET penalties overcome this difficulty by contracting some coefficients to zero, in lean methods that result in the automatic selection of variables as we will discuss later (James et al., 2013). Further information on use cases, pros and cons of early integration methods, can be found in Supplementary Table S2.

## 3 Data integration approaches

The vast majority of multi-omic integration approaches are founded upon statistical modeling, classification and feature selection methods. In this section we will present some of the most successful algorithms to reduce dimensionality and extract relevant information allowing for integrated models in multi-omics.

### 3.1 LASSO and related approaches

#### 3.1.1 LASSO

The *Least Absolute Shrinkage and Selection Operator* (LASSO) is a type of regularization technique used for regression models, aimed at both improving prediction accuracy and interpretability. Such regularization adds a penalty to the regression coefficients proportional to their absolute values. This has the effect of shrinking some coefficients to zero, effectively performing variable selection. For this reason, LASSO is widely used to handle overfitting in models with many predictors and to identify a subset of predictors that have the most significant impact on the response variable.

LASSO penalty has been added to various statistical methodologies, resulting in the concise (i.e., sparse) versions of analyses such as PCA, JIVE, PLS, and RGCCA. Its ability to select variables has been exploited to suggest possible regulators of gene expression, biomarkers, and, as desired here, to propose relationships between different functional levels. Let us examin this in more detail.

The difference between the LASSO and ridge penalties is the object on which they work. While the ridge penalty scales the $l_{2}$ norm of the coefficient vector, LASSO transforms the $l_{1}$ norm, using the same λ parameter with values between 0 and 1 that is chosen by cross-validation (CV). The difference between these norms is a power, as the $l_{1}$ norm of a vector is defined as the sum of the absolute values, while the $l_{2}$ norm involves the sum of the squares (James et al., 2013). Outside of this small difference in definition, the mentioned statistical analysis methods remain largely unchanged in terms of goals and assumptions.

Without necessarily using partial least squares fitting, regression analysis with LASSO penalty has achieved the previously ignored goal of identifying potential regulatory elements. An example is the use of miRDriver to suggest miRNAs that extend the effect of a copy number alteration to their trans targets. Using the TCGA breast cancer data, it can be seen that miRDriver tends to select miRNAs related to cancer and with prognostic value, such as miR-1224, miR-31, let-7 g, and let-7b (Bose and Bozdag, 2019). Another example is the asymmetric integration of DNA methylation, CNVs, miRNAs, and TF binding sites with differentially expressed genes in breast cancer subtypes. Comparing the prediction capacity of the omics separately and together, the integrated models had better performance, with a significant increase when adding methylation data. Although there are shared regulators among the four subtypes, including E2F1 and CITED2, the possible regulatory mechanism is not developed, but rather the stratification capacity of a transcriptional signature with basal subtype regulators is proven (Huang et al., 2019).

Going further, possible regulatory mechanisms have been proposed based on this type of regression. Setty et al. modeled differential expression between glioblastomas and normal tissue, with a LASSO regression that integrates promoter methylation and average copy number, with the count of TFs and miRNAs that can bind to the regulatory region. The copy number was selected by the LASSO in all cases, while the methylation coefficients were always kept as large negative values. The variability of the coefficients corresponding to miRNAs can be explained by a low correlation with the expression of the target gene, due to the simultaneous action of other regulators or, as has been suggested before, because miRNAs only modestly affect gene expression. When the key predictors were searched by subtype and gene, using a dependency analysis, it was observed that REST, a repressor of neuronal genes in non-neuronal cells, is key in the different subtypes. Although the interactions shown there come from the literature, the regulatory model depends on the selection of REST and miR-124 in the regressions of all subtypes, and the selection of YY1 and miR-132 in one of them (Setty et al., 2012).

Similarly, Li et al. integrated CNVs, methylation, and microRNAs to predict ovarian cancer data from TCGA. The decomposition of the matrices yields factors of, on average, 45 CNV loci, 43 CpG sites, 5 miRNAs, and 44 genes. While the correlation between CNV events and expression levels remains positive, the correlation with methylation varies. When deriving correlation networks from the co-selected elements, the authors emphasize that the elements would remain isolated if it were not for the integration. Although these networks do not necessarily reflect causal relationships, they can serve as a starting point for studying the underlying mechanisms (Li et al., 2012).

Without focusing on any mechanism, integration has also been used to build multi-omic networks, from which general conclusions can be drawn regarding the importance of different functional levels in cancer. Sohn et al. built networks of CpGs, miRNAs, and CNVs with the coefficients of a predictive model of gene expression in ovarian cancer. The integrated model predicts expression better than single-omic models. Patterns of highly expressed genes have as their preferred predictor the alteration in the number of copies; while genes with greater variability are better explained with methylation, which would indicate a dynamic effect of DNA methylation regulation, which is increasingly supported by evidence (Rossnerova et al., 2020). As for the networks, the network of the integrated model has greater modularity and enriches for more specific functions than single-omic networks. Edges with higher weight (coefficient) tend to involve methylation sites and as the weight decreases the proportion of CNVs increases, while miRNAs remain stable (Sohn et al., 2013).

Although they only integrate data from DNA methylation and breast cancer expression, Lee et al. built networks with the coefficients of LASSO regression; implementing a weighted kernel to share information between all samples, without failing to obtain specific coefficients for each subtype. The use of the kernel improves the predictions of genes linked to some subtype and especially to the enrichment of Her2, which only has 16 samples. For each target gene, they only consider the CpG sites of the pathways in which the gene participates and the model selects between 200 and 300 sites, which form networks with 88.82% of the edges in common between subtypes. The most connected CpGs participate in cancer progression, as happens with LEP and FGFR3 in the luminal B subtype. The best-predicted genes encode GTPases, transcription factors, and DNA binding proteins. By adding the error rates of the genes in a pathway, it is possible to estimate the impact of methylation on the pathway, leaving in the top of prediction of the four subtypes pathways of carbohydrate metabolism such as glycolysis/gluconeogenesis, the pentose phosphate pathway and fructose and mannose metabolism (Lee et al., 2017).

Finally, LASSO selection has also been used to filter potential therapeutic targets from among multiple genomic elements. This was possible by adjusting a streamlined multivariate Cox model of ovarian cancer expression data, miRNAs, DNA methylation, and copy number alteration. The resulting signature contains 156 elements and predicts progression-free survival better than larger signatures based on a single omics. The integrated signature is enriched for genes involved in immune response and metabolism. By ordering the elements of the signature by their ability to stratify patients, it is possible to further filter the list of potential biomarkers (Mankoo et al., 2011).

Although dimensional reduction alone may be sufficient to predict phenotypes and group samples, LASSO penalization facilitates the identification of potential regulators and the construction of concise networks and, in doing so, opens the possibility of fulfilling the early integration promise of discovering multi-omic mechanisms. The major problem with LASSO penalization is the instability of the selected variables. This is an inherent problem and has a well-established palliative, based on the frequency with which variables are selected in random subsets of the data. Frequencies can be summarized with the Fleiss score, which reflects concordance between subsets and takes larger values as stability increases. You can also simply choose a cut-off point on the selection frequency (Bose and Bozdag, 2019; Bravo-Merodio et al., 2019), which however adds arbitrariness. Regardless of the chosen strategy, highly correlated variables are more likely to be selected in each subset (Lê Cao et al., 2011).

In addition, LASSO penalization has shown limitations when there are groups of strongly correlated variables within the same block, in which case LASSO tends to choose only one variable. In situations where there are more samples than variables, *ridge* penalization performs better. In situations where there are more variables than samples, as is the case with omics, LASSO can only select as many variables as there are samples. An ideal method should eliminate trivial variables and include complete groups as long as one of the variables is selected. With this in mind, an intermediate penalization between ridge and LASSO, known as elastic net, was proposed (Zou and Hastie, 2005).

#### 3.1.2 ENET

Elastic net, ENET for short, combines the two penalties, *ridge* and LASSO, to obtain a method that contracts the regression coefficients to zero; but also filters complete groups of correlated variables. Due to the selection of groups, elastic net selects more variables than LASSO and often achieves more accurate predictions, especially in the presence of collinearity.

In its simplest derivation, elastic net penalty consists of the sum of *ridge* and LASSO penalties, so it involves two parameters, $\lambda_{2}$ which acts on $l_{2}$ and $\lambda_{1}$ to scale $l_{1}$ . To simplify, the λ parameters are replaced by an α value, which converts the penalty to:

$$\left(1-\alpha \right)|\beta_{1}|+\alpha |\beta {|}^{2}\leq t;\alpha =\frac{\lambda_{2}}{\lambda_{2}+\lambda_{1}}$$

This way, when the mixing parameter, α , is 1, the ENET becomes the ridge penalty and when α is 0, it becomes the LASSO. Therefore, the ENET exists between 0 and 1, although the evidence indicates that the best performance is achieved with values very close to either of the two extremes. Being a strictly convex function for any value greater than 0, identical predictors receive the same coefficient, which guarantees the grouping of strongly correlated variables. The similarity with LASSO allows for efficient computation and its application to solve both regression and classification problems (Zou and Hastie, 2005).

Thus, the mixing parameter controls how sparse the model is and is chosen by cross-validation (CV). As with the other penalties, CV based on small data sets, smaller than 100 samples, produces excess variance that can be problematic. Similarly, the values of the coefficients have no relation to the original measures, which makes their interpretation difficult (Kirpich et al., 2018). Finally, in both penalties, the variables lack a measure of significance that supports their selection (Pineda et al., 2015). The difference between LASSO and ENET lies in an advantage of elastic nets when there is an excess of relevant predictors or when there are groups of correlated variables. Otherwise, when the models are highly sparse, the performance of both penalties is equivalent (Neto et al., 2014).

The superiority of the elastic net increases with the number of samples and the level of correlation; but it declines after a while when the number of predictors increases (Neto et al., 2014). Counterintuitively, false positives also increase with more samples, fewer predictors, and higher correlation, which is controlled by raising the value of α . It is on this basis that the value of 0.5 for the mixing parameter is recommended, as it controls the type I error and tends to filter out complete groups of correlated variables (Kirpich et al., 2018).

Instead of choosing one penalty over the other, the use of both has been proposed for the robust identification of markers. Based on three cohorts with lipidomic data, horizontal integration with the two penalties yields a marker and a classification of subjects based on that marker, which surpasses differences between cohorts. Testing the same combined scheme with data on pancreatic adenocarcinoma expression and, separately, on myeloid leukemia, the recovery of highly discriminatory genes is repeated, which, when connected in a network of interactions, model the differences between cancer and normal tissue (Bravo-Merodio et al., 2019).

In addition to joint use, Pineda and company proposed a permutation strategy in order to evaluate the significance of the selection. The two penalties were used to predict bladder cancer expression from genetic variants, methylation, or a combination of both omics. The result of LASSO is a selection of 9 genes significantly explained by genetic variants, 19 by CpGs, and 23 by the combined model. Contrary to expectation, the selection by the elastic net is smaller, with 11, 6 and 4 genes, respectively. Although the intersection between penalties is small, it is taken as additional evidence of the model. Notably, the genes selected by both LASSO and ENET achieve similar *p*-values (Pineda et al., 2015).

Another innovation is the adjustment of different penalties by omics, which Liu et al. implement to classify samples of acute myeloid leukemia and, independently, prostate adenocarcinoma, based on the integration of gene expression and DNA methylation data. The idea is that forcing uniform contraction over omics with different sizes and different magnitude effects can unfairly punish important but more subtle variables than the entire block of the other omics. Then, the adjustment of different penalties addresses the problem of early integration with compatibility between platforms. Although this adds a parameter to adjust per omics, which requires more computing time, it does not change the underlying method, as the programming packages that contain the elastic net, such as glmnet and caret, usually already include this option. Thus, the authors fix α at 0.5 and optimize a λ contraction value and a κ contraction radius, which ultimately improve the model’s predictability. Simulated data indicate that the greater the contrast in the number of variables between the blocks, the greater the differential penalty should be, loading the contraction of coefficients on the omics with more noise. AML classification improves when the penalty affects the methylation data less, as chromosomal aberrations cannot be identified only with expression. On the other hand, prostate tumor classification does not improve with differential penalty, but the optimal κ is slightly above one and selects an excess of transcripts, mostly already linked to cancer (Liu et al., 2018).

Due to its origin, the elastic net shares difficulties with LASSO. Namely, cross-validation parametrization, the interpretability of the coefficients, and their lack of significance. Fortunately, each of these issues has, if not complete, well-established solutions, such as the use of alternative *k-folds*, *leave-one-out*, or *bootstrapping* in the case of parameter choice or the obtaining of empirical values, as Pineda et al. did (Pineda et al., 2015), to address the lack of significance values. Although the coefficients are not comparable to the original values of the omics, Huang and collaborators (Huang et al., 2019) show how the coefficients reflect the strength of the association between predictors and even give them a sign. As tools for early integration, both multivariate sparse methods face differences between platforms, either with normalizations that control the weight of each omics (De Tayrac et al., 2009) or by varying the penalty to which they are subjected, as Liu and coworkers demonstrated (Liu et al., 2018).

Finally, by depending on dimensionality reduction methods, they inherit the issues of matrix decomposition. Since latent components are linear combinations of the original variables, only relationships with an extended linear effect on different predictors can be captured, when probably the effect of alterations is not concentrated on a single factor (Rohart et al., 2017; Vlachavas et al., 2021). Additionally, the number of components limits the information captured. Although the first components explain the majority of the variance and the last ones would be linked with noise; cancer data encompasses tissue signals, exposure to mutagens, treatment, and immune infiltration, among others, which can increase the amount of necessary components. Even more, the components of the sparse models, with most coefficients at zero, explain a lower percentage of variance (Lê Cao et al., 2008). When it comes to classifying samples, the convention is to recover K-1 components, where K represents the number of classes (Lê Cao et al., 2011). However, when seeking multi-omic mechanisms, there are no classes to guide us.

It has been suggested that dividing the data blocks into functional pathways or sub-blocks can simplify the choice of the number of components, by giving them a more direct interpretation (Garali et al., 2018). Since both sparse methods end up in correlation networks, it is also relevant to highlight that these networks fail to distinguish between direct and indirect relationships, which could be resolved by estimating the precision matrix (Fan et al., 2020). Although the precision matrix is nothing but the inverse of the covariance matrix, its acquisition is not a simple process when there are co-linear blocks or more predictors than observations (Garali et al., 2018). Therefore, when implementing a sparse multivariate model, it is necessary to weigh the aforementioned disadvantages against the ability to integrate large amounts of variables without the strict need for prior filtering (Pineda et al., 2015).

On the issue of how these methods are able to deal with the complex, heterogeneous nature of cancer at the molecular scale it is relevant to notice that under some circumstances LASSO, in particular, may not be the best choice to accommodate cancer related heterogeneity. ENET, on the other hand, may be more robust. This is so since it combines the l1 penalty of LASSO and the l2 penalty of Ridge Regression, allowing it to perform variable selection (like LASSO) while also handling multicollinearity (like Ridge Regression), this makes it more robust in situations where there are groups of correlated predictors, as it can select entire groups of correlated variables rather than just individual variables. ENET will work in two ways under such circumstances:

1. The l1 component of Elastic Net encourages sparsity, meaning it can select a subset of important predictors, which is useful in heterogeneous data where some variables may be irrelevant.

2. The l2 component helps to stabilize the solution when predictors are highly correlated, which is common in heterogeneous data.

Heterogeneous data often have multicollinearity issues due to the presence of similar or redundant features. The l2 penalty in Elastic Net helps to mitigate these issues by shrinking correlated predictors together rather than forcing the model to select only one from a group. Hence ENET’s ability to select groups of correlated variables makes it particularly useful for data with inherent group structures or when predictors are naturally grouped together due to the nature of the data. We have to consider however that Elastic Net requires tuning two regularization parameters, $\lambda_{1}$ and $\lambda_{2}$ , which control the balance between l1 and l2 penalties. Cross-validation is typically needed to find the optimal values. For very large datasets this may become computationally burdensome.

One also has to consider, however, that both LASSO and Elastic Net can produce false findings when applied to multi-omics data because these methods assume a uniform error distribution and may struggle with long-tailed distributions or data contamination. Additionally, they do not inherently account for the complex correlations and network structures often present in multi-omics data, leading to potential inaccuracies in identifying relevant associations and in variable selection. Interestingly, to improve upon this, Wu and collaborators Wu et al. (2018) developed a robust approach to better address these challenges in the context of joint copy number variant and gene expression concurrent data.

In brief, the method proposed (termed *Robust network-based penalized estimation*) consists in the following: 1) Using a partially linear model with a nonlinear cis-acting CNV effect for each gene expression (GE). 2) Developing a robust loss function to accommodate the effects of long-tailed distributions and data contamination. 3) With this penalization approach addressing high dimensionality and identifying relevant CNVs. 4) Introducing a network structure to account for correlations among CNVs, 5) Developing an effective computational algorithm and rigorously establishing consistency properties. This approach aimed to improve prediction accuracy, stability, and biological plausibility in multi-omics data analysis. More information regarding integration approaches based on LASSO/ENET can be found in Supplementary Table S3.

### 3.2 Canonical correlation analysis and partial least squares

In the case of SGCCA–where the “S” stands for sparse–, the goal of RGCCA to extract shared information between blocks of data is maintained for each of them, that is, not a single shared structure is obtained as with JIVE, but rather a latent component is generated for each block. This block component summarizes the variance of its own data while being correlated with other blocks. The correlated blocks depend on a C parameter (more on this function below). In addition to being regularized as sparse, SGCCA has as its main characteristic the maximization of covariance. While RGCCA can maximize covariance, correlation, or a compromise between the two, SGCCA only optimizes covariance, prioritizing finding block components that contain the highest possible variance and, as a second priority, recovering correlation with neighboring components.

Thus, SGCCA is defined with the optimization problem:

$max_{w_{1},\ldots ,w_{j}}\sum_{j,k=1}^{J}c_{jk}g\left(cov\left(X_{j}w_{j},X_{k}w_{k}\right)\right)\text{ s.t. }\left\{\begin{array}{c} \|w_{j}{\|}_{2}=1\\ \|w_{j}{\|}_{1}\leq s_{j} \end{array}\right.,j=1,\ldots ,J$

Where:

$$\begin{array}{ll} X_{1},\ldots ,X_{j} & =\text{Data blocks}\\ w_{j} & =\text{Coefficient vectors}\\ X_{j}w_{j} & =\text{Block components}\\ g & =\text{Convex continuous function allowing different optimization }\text{criteria such as:}\\ & \text{⋅}\text{ identity function which simply optimizes covariance}\\ & \text{⋅}\mathrm{Horst}\text{function, which penalizes negative correlation among blocks}\\ & \text{⋅}\text{ centroid function allowing negative correlations}\\ C & =\text{Square matrix whose size is given by the number of blocks, values }1\text{ and 0,}\\ & \text{depending on whether the blocks are connected or not}\\ s_{j} & =\text{positive constant that determines penalization degree, that is}\\ & \text{how sparse is w } \end{array}$$

The (Tenenhaus et al., 2014; Garali et al., 2018) C matrix allows for examining different relationships between functional levels. In the original publication of SGCCA, three C designs were studied by Tenenhaus and collaborators with respect to the relationship between gene expression, chromosomal imbalance, and the location or subtype of pediatric gliomas. The design with the three blocks connected seeks simultaneous alterations of the two functional levels with respect to the subtypes. When the connecting point is location, the objective is the alterations associated with the subtypes, regardless of the relationship between expression and imbalance. Finally, placing expression as the bridge between the other two blocks speaks of imbalances that affect expression and in turn affect the subtype. The second design had the best predictive values, confirming that in that case the aim was to determine the subtypes, although the expression block yields more discriminatory information than the imbalance block. A smaller number of variables are also reported to be selected compared to comparable methods and low sensitivity to the g function (Tenenhaus et al., 2014).

Just as Principal Component Analysis (PCA) and Partial Least Squares regression (PLS), can be derived as special cases of RGCCA (Garali et al., 2018), the sparse version of PLS, sPLS, is based on SGCCA. There are however, some interesting differences between these approaches: PLS is primarily used for regression and classification tasks, especially when dealing with highly collinear and high-dimensional data. PLS works by projecting the predictors and the response variables into a new space and finding a linear relationship in this projected space. It creates components (called latent variables) that are linear combinations of the original predictors, with the goal of maximizing the covariance between the predictors and the response.

PCA aims to reduce the dimensionality of a dataset while retaining as much variability (information) as possible. It works by performing noise reduction by keeping only the components with significant variance. For this reason, PCA is used for feature extraction and preparation for other machine learning algorithms.

In contrast, the mixOmics package in R has implemented instances of Tenenhaus’s SGCCA specifically designed for both vertical integration and horizontal integration and, especially, supervised extensions dedicated to classification and prediction. In doing so, it strongly relies on the robustness of the Canonical Correlation Analysis (CCA) family of methods. CCA is used to understand the relationships between two sets of variables. It aims to find linear combinations of variables in each set that are maximally correlated with each other. It is particularly useful in multi-view data analysis (the case of multi-omics), where two sets of variables (e.g., different data modalities) are measured on the same set of observations.

For usability, mixOmics replaces the penalty parameter with the number of elements to recover from each dimension and facilitates its adjustment with functions taken directly from statistical research. At the same time, the package captures measures of error and stability of the selected variables, allowing for efficient visualization. When examining its own tools, the authors report higher discriminatory capacity of the subtypes of breast cancer in the integration of transcriptome and proteome data compared to using miRNA expression data, which, however, are strongly correlated with the transcriptome (González et al., 2012; Rohart et al., 2017; Singh et al., 2019).

The advantages of sPLS include greater stability with collinear data - which is common in omics - compared to other types of regression or even canonical correlation analysis (CCA) itself; explained variance similar between different levels of penalization; orthogonality of latent factors within the same block, but not of factors from different blocks - which arise from the maximization of covariance - (Rohart et al., 2017); superior performance to classification tools such as random forests and nearest centroids; and 4 different modes of decomposing the data matrix, with different purposes. The regression mode, or PLS2, aims to explain Y (e.g., dependent features) from X (independent variables), so the decomposition is asymmetric and the obtained latent factors will not be the same as when predicting X with Y . The classic mode is identical to the regression mode and is also identified as PLS2, but uses a different normalization, which produces different Y coefficients. The canonical mode, on the other hand, is symmetric, as its goal is to model the relationships between blocks, without assuming a direction in them. As an exploratory mode, it cannot be subjected to the same adjustment criteria as the supervised alternatives. Finally, the invariant mode would also be asymmetric, as it does not decompose the response but rather performs a redundancy analysis of X with respect to Y (Lê Cao et al., 2008; Lê Cao et al., 2011).

Coupling mixOmics’s sPLS and MOGSA, Chapell and colleagues do not suggest a possible multi-omic mechanism, but rather a regulatory axis that would be desirable to intervene. By integrating data from basal breast cancer cell lines, which include DNA methylation, gene expression, protein expression, phosphoproteomics, and histone modification, TGFB1, TGFBR2, KLF6, KLF12, PIK3R3, VIM, NES, RASL11B, HOXC9, LAMB3, PRKCD, PRKCE, and MELK were recovered in the first two latent components. Upon further review, the authors find that TGFb signaling differs effectively between lines with and without BRCA1 mutations and that its enrichment depends on the phosphorylation of SMAD5. Since in tumor cells the stimulated TGFb receptor phosphorylates SMAD1/5 and promotes cell migration, it is concluded that the TGFb-TGFBR1-SMAD1/5 axis could be of clinical interest (Chappell et al., 2021). More information about PLS/CCA integration approaches can be found in Supplementary Table S4.

## 4 Network integration

In his 2016 review, Bersanelli hierarchically divided integrative methods by distinguishing methods based on networks from those independent of them (Bersanelli et al., 2016a). As seen, multivariate methods can generate multi-omic networks, but they do not need them to concretize integration, but rather work at the level of the data matrix. On the other hand, there are tools that exploit the ability to find dependency relationships between any pair of random variables, converted into nodes (Hawe et al., 2019), such as the expression of a miRNA and the methylation of a CpG.

Moving forward by pairs, the relationships are blind to the effect of others and end in dense networks of limited interpretability (Hawe et al., 2019). Therefore, an extra step is needed, which focuses on interactions that meet certain criteria, restricting themselves to interactions with support in the known.

It is also possible to estimate conditional dependencies (partial correlations) through Gaussian graphical models, which obtain the network from the precision matrix (Hawe et al., 2019). However, this approach is still being explored and will only be touched upon in the context of DRAGON (Determining Regulatory Associations using Graphical models on multi-Omic Networks). Since omics produce data matrices with more predictors than observations, the covariance matrix is not invertible and cannot be used to calculate partial correlations. DRAGON introduces a penalty that contracts the covariance matrix and allows the obtaining of a contracted precision matrix. In this way, it has become a tool that is still being evaluated, but with promising results, such as the identification of ELF4 and ZBTB33, two co-expressed TFs due to their co-methylation, in TCGA breast cancer data (Weighill et al., 2021).

While the connection of any pair of variables generates multipartite networks, multi-omic integration is also possible through multi-layer networks. Multipartite networks have as many types of nodes as integrated omics and the edges represent both intra and inter-omic relationships. In contrast, multi-layer networks represent relationships within the same set of nodes through different functional levels, being then a compilation of the networks derived from each omics (Bersanelli et al., 2016a). The multiple links connecting a pair of nodes in this formalism are the basis of layered integration algorithms.

Additionally, there are examples of integration that do not fit into any of the sections. Such is the case of the division of cancer types into those characterized by mutations and those linked to the number of copies. This division arises from the analysis of the modularity of a bipartite graph, of patients and alterations, and places breast cancer in the group associated with CNVs. Although the network involves CNVs, point mutations, and DNA methylation, effectively integrating different omics; the nodes are not random variables, but isolated entities that connect to the network according to the presence or absence. The network also does not match the multi-layer formalism, as the nodes only exist in their own functional level, which would be that of alterations in cancer. However, by ignoring the relationships between alterations, which only connect through co-occurrence in a patient and involve a filtering of complete omics to frequent events, this work qualifies as intermediate integration. Despite the relatively simple strategy, the network approach allows such a powerful conclusion as suggesting different oncogenic mechanisms for tumors, from different tissues, that group around specific combinations of functional events (Ciriello et al., 2013).

Another example is the identification of transcription factors that are sensitive to DNA methylation in most cancer types. In this case, it all starts with a gene expression regression network, which links TFs and genes, and the integration is more of an incorporation of methylation patterns into the attributes of the genes, to evaluate the effect of epigenetics on the TF-gene relationship. Comparing the different types of cancer, they found that only 0.28% of the regulatory relationships mediated by methylation appear in more than 4 types of cancer, yet there are TFs that consistently are sensitive to methylation. These TFs regulate more targets, show differential expression, and are enriched in signal transduction pathways, cell adhesion, and the ETS family (Wang et al., 2020). Here, the nodes do represent random variables, but from the same omics, and the network is essential for the joint interpretation of the expression and methylation data, which is the final objective of the integration. Additionally, this debatable example of multi-omic integration by networks, considers the effect of one functional level on the other and focuses on the interactions, first to recognize those that depend on DNA methylation, and then to contrast the different subtypes.

As disparate as these examples may be, they highlight the importance of connections, whether between TFs and genes or between tumors through alterations and therefore, align with network biology, this systems biology approach focused on inferring network models on biological phenomena and their analysis using graph theory. In particular, the examples presented here belong to the probabilistic approach of network biology. This is a top-down approach, which builds the model to be tested from massive data, not just from the (limited) information, already stored in the databases (Hernández-Lemus, 2014). Next, we will delve deeper into this probabilistic approach and its implications for multi-omic integration.

### 4.1 Probabilistic networks

Most gene network reconstruction methods, start with a single omics dataset –usually gene expression–, used to suggest mechanisms from the relationships between genes. Think about the culprits by association in interaction networks (Lee et al., 2011). The difference with genetic regulation networks is that the edges reflect statistical dependencies between expression patterns. Two genes connected by their expression patterns may hold a functional relationship or simply co-occurrence (Margolin et al., 2006). The difference with multi-omic networks is that the variables exist on different scales and need to be normalized. If the measure of statistical dependency is correlation, it’s not enough to have comparable values, the variance distributions must also be similar (Tarazona et al., 2020).

Although Pearson’s correlation may be the most common measure, there are multiple tools that use, fort instance, mutual information (MI). Mutual information is a measure of statistical dependence that comes from information theory, and by treating information as a reduction of uncertainty, it measures the reduction in uncertainty of one variable with respect to the information about another variable (Mousavian et al., 2016). MI is indeed the maximum entropy/maximum likelihood non-parametric measure of statistical significance.

$$I\left(X,Y\right)=\underset{x\epsilon X}{\sum }\underset{y\epsilon Y}{\sum }p\left(x,y\right)log\frac{p\left(x,y\right)}{p\left(x\right)p\left(y\right)}$$

$$I\left(X,Y\right)=H\left(X\right)-H\left(X|Y\right)=H\left(Y\right)-H\left(Y|X\right)=I\left(Y,X\right)$$

Mutual information has an advantage over correlation in capturing non-linear relationships, being insensitive to parameterization, and able to be estimated quickly, all desirable characteristics for multi-omic integration, with non-linear relationships, different scales, and a large number of variables. On the other hand, mutual information is always positive, even when evaluated on random patterns, so it is necessary to establish empirical thresholds (Margolin et al., 2006). Additionally, it is a symmetric measure, which does not allow giving direction to interactions (Hernández-Lemus and Rangel-Escareño, 2011).

In the so-called relevance networks, mutual information is calculated similarly and all pairs below a threshold are discarded. Although this strategy highlights functional nodes, it is unable to distinguish direct and indirect relationships. Thus, the ARACNE (Algorithm for the Reconstruction of Accurate Cellular Networks) was built on relevance networks, which finds irreducible, probably regulatory, statistical dependencies by getting rid of indirect interactions. ARACNE’s DPI (Data Processing Inequality) examines each triangle in the relevance network and discards the edge with the least weight, considering that mutual information decreases rapidly as the distance between nodes increases (Margolin et al., 2006).

ARACNE has been exploited for integrating miRNA and transcript expression data in breast cancer. Comparing networks of cancer and adjacent normal tissue, Drago-Garcia et al. observed a reduction of links between miRNAs and transcripts in cancer, despite the fact that there are more miRNAs, which connect to each other and are essential for maintaining network cohesion. They also report an enrichment of processes related to the immune system and cell adhesion, as well as a very clear association between the miR-200 family and the DKL1-DIO3 cluster, which participates in epithelial-mesenchymal transformation. Regarding the technique, the authors find quantitative differences in the distribution of MI between miRNAs and transcripts, which may reflect the differences between the molecules and foreshadow difficulties in distinguishing direct interactions in the multi-omic approach (Drago-García et al., 2017).

The solution for discriminating between direct and indirect relationships may be conditional mutual information. If the conditional information between a pair of nodes, with respect to a third, is less than a threshold, then the edge connecting the pair should be removed (Mousavian et al., 2016). In this way, Liu and colleagues built networks for different types of cancer, based on mutual information between transcripts and TFs, given the promoter methylation pattern and copy number. Then, to examine the regulatory effects, they adjusted linear regressions that show better predictability by integrating the omics than using only expression data, genes that strongly depend on copy number and genes that mainly depend on methylation. These latter ones overlap between cancer types and are associated with tumorigeneisis. Finally, they used promoters whose methylation affects transcription to group cancer types and examined the survival curves of the groups, identifying 10 types of cancer, including breast cancer, where methylation is an important determinant of tumor aggressiveness. Despite the approach yielding highly interesting results, the authors warn that it requires large sample sizes and data with sufficient variance to find reliable networks (Liu et al., 2019).

In turn, the lack of direction of the edges can be resolved by switching from searching for regulators for each gene, to searching for regulators for groups of genes co-expressed through different conditions. LeMoNe, later LemonTree, begins with a two-way clustering, which combines co-expressed genes with high probability into enriched clusters of specific functional categories (Joshi et al., 2007). As a second step, the algorithm sorts a list of possible regulators, according to their ability to predict the expression of the cluster in different conditions. Although only TFs, miRNAs and CNVs have been tested separately as regulators, there are no formal restrictions on the type of variables that can be integrated. In the case of miRNAs, most are assigned to a single group, but there are repeated miRNAs between groups. In the case of CNVs, LemonTree produces modules with more significant enrichments than the dedicated tool CONEXIC (Bonnet et al., 2010; 2015). This example proposes a solution to the problem of symmetry in statistical dependency measures, such as MI and correlation, and successfully identifies potential regulators for groups of genes associated with functions, but in the process erases the boundaries between regulators and ignores their possible interactions.

#### 4.1.1 Uncertainty in probabilistic network integration

The probabilistic component of network modeling introduces a powerful framework for capturing the inherent uncertainty in biological systems, which is particularly relevant when integrating multi-omics data. Probabilistic models, such as Bayesian networks and Markov random fields, represent the relationships between variables as probabilistic dependencies, allowing for the quantification of uncertainty in the predicted interactions (Vahabi and Michailidis, 2022; Subramanian et al., 2020; Bersanelli et al., 2016b). This is especially important in biological contexts where data is often noisy, incomplete, and subject to various sources of variability. By modeling the dependencies probabilistically, these approaches can incorporate prior knowledge, handle missing data, and provide more robust predictions that reflect the uncertainty associated with the biological processes being studied (Miao et al., 2021; Wang W. et al., 2021; Sathyanarayanan et al., 2020).

Uncertainty quantification in probabilistic models is typically achieved through the estimation of probability distributions over the possible states of the network components (Graw et al., 2021; Athieniti and Spyrou, 2023). For example, in a Bayesian network, the relationships between variables are represented by conditional probability distributions, which describe how the probability of one variable depends on the state of other variables (Mallick et al., 2024; Fang et al., 2018; Wang et al., 2019). The uncertainty in these relationships can be quantified by the width of these distributions, indicating the level of confidence in the inferred interactions. In practice, this allows researchers to not only predict the most likely interactions but also to assess the degree of uncertainty associated with each prediction, which can be critical when making decisions based on these models, such as identifying potential drug targets or biomarkers (Nicora et al., 2020; Wang R.-S. et al., 2023; Wang Y. et al., 2021).

In the context of multi-omics data integration, incorporating probabilistic modeling allows for a more nuanced understanding of the complex interactions between different omic layers. For instance, when integrating genomics, transcriptomics, and proteomics data, a probabilistic approach can account for the uncertainty in how changes at the genomic level translate to changes at the transcriptomic and proteomic levels. This is particularly valuable given the often non-linear and context-dependent nature of these interactions. By quantifying uncertainty, probabilistic models can help to identify which relationships are robust and likely to hold across different datasets and conditions, and which are more tentative and may require further validation (Yin et al., 2022; Yang et al., 2020).

The effect of uncertainty quantification on integration methods is profound. It can enhance the interpretability of the integrated models by providing a measure of confidence in the predicted interactions, helping researchers to distinguish between strong, well-supported findings and those that are more speculative. This is particularly important in translational research, where the consequences of acting on uncertain or incorrect predictions can be significant. Furthermore, probabilistic models can be used to prioritize experimental validation efforts, directing resources towards investigating the most promising hypotheses that emerge from the integration process.

Moreover, probabilistic modeling facilitates the integration of prior knowledge, such as known biological pathways or regulatory mechanisms, into the analysis. This prior information can be encoded as prior probability distributions, which are then updated as new data is integrated. This Bayesian updating process allows the model to refine its predictions as more data becomes available, continually improving the accuracy and reliability of the integrated network. This dynamic aspect of probabilistic modeling is particularly valuable in the fast-evolving field of omics, where new data is constantly being generated. A comparative table of probabilistic network approaches is presented in Supplementary Table S5.

The algorithms described here infer potentially regulatory networks from complete omics. The resulting graphs are not complete, because some edges do not pass thresholds if we are talking about MI networks or conditional MI, or because there is an initial restriction on who is joined to whom in LeMoNe/LemonTree. Once the graphs are obtained, the focus of the analysis can be narrowed even further around nodes with important roles in the network. As a result of this, there is an indirect filter on the nodes, only the connected ones appear, so that the perspective of the analysis is guided by the data itself. On the other hand, there is a whole set of tools guided by interactions already deposited in databases, as detailed below.

### 4.2 Networks known a priori

Instead of relying completely on statistics, multi-omic data can be organized based on known interactions, thus preserving proven edges that otherwise might be diluted in accumulated noise (Koh et al., 2019). By simply retaking what is known, this approach is not useful for proposing multi-omic mechanisms, because the relationships between different functional levels are not known, at least not to the degree of specificity required. On the other hand, by focusing on already established information, it leads the discussion directly to functions and genes, without getting lost in the sea of new data. In other words, the integration guided by known networks is not the approach to finding novelties but rather to put the already collected information to use, in the form of proposed markers or signatures and therapeutic targets. Additionally, since this approach does not necessarily depend on statistics, the sample size becomes less important, and it is even possible to talk about a network for each patient.

The simplest applications of this approach only map altered genes to the network and record topologically important nodes, such as hubs and bottlenecks. APODHIN (Analysis of Pan-omics Data in Human Interactome Network) sums the logFC values of the different omics, if it is a vertical integration, or cohorts, for horizontal integration, and reports the associated pathways, in addition to the topologically important genes (Biswas et al., 2020). Instead of altered genes, Cava et al. map genes with prognostic ability of basal breast cancer. The innovation here is double, because first, they extract a subnetwork per patient, using the prognostic genes that also have an alteration in the number of copies in that patient and then look for drugs linked to the five genes with the highest degree, turning a simple analysis into a tool for personalized medicine. In basal tumors, the simultaneous alteration of BRCA1 and TP53 is observed in half of the cases, both being central nodes in the network, potentially susceptible to various drugs, and thus desirable therapeutic targets (Cava et al., 2021).

The most sophisticated applications calculate some type of score by taking advantage of the network. Such is the case of NetICS (Network-based Integration of Multi-omics Data), which seeks out mediating genes that connect the alterations with the effectors, based on the functional interactions deposited in databases, such as KEGG and miRTarBase. Since the networks a priori have direction, it is enough to diffuse downstream the disturbance score and upstream the differential expression score of the effectors, using a heat diffusion algorithm, to obtain a mediation score. After repeating the process for each sample, the combination of the scores generates a global value. In breast cancer, the mediating genes are enriched in signaling pathways. EP300 and TP53 stand out, each downstream of 5 different genes, altered in 50% of the samples, and connected through the effectors. The major disadvantage of NetICS is a bias towards highly connected genes, which by chance, have a greater probability of having altered genes as neighbors (Dimitrakopoulos et al., 2018).

Similarly, data from GWAS, eQTLs, mQTLs, ATAC-seq, and the annotation of the Roadmap project have been integrated to propose key genes. The process starts by connecting genes with the SNPs that affect them, directly or indirectly, and locating those genes in two different networks of protein-protein interactions (PPI), a general and a tissue-specific one. For each of the affected genes, functional and prognostic signatures enrichment must be verified, such as MammaPrint and PAM50, among the first neighbors and obtain a combined score of prognostic enrichment in the two networks. By calculating this score for breast cancer, it can be concluded that SNPs affect cancer and signaling pathways, such as MAP kinases, TGF-beta, and WNT. The 20 genes with the highest prognostic enrichment include known cancer genes and novelties such as RNASEH2A, which exhibits abnormal expression in tumors and is associated with copy number alterations, lower survival, and ER-tumors (Chen et al., 2020).

Mergeomics promises to integrate any set of omics, following largely the same steps as in the previous example. First, it evaluates the enrichment of any predefined set of genes, in any type of disease association data (GWAS, TWAS, EWAS) and if there are different types it calculates a meta-value of significance. Later, the enriched sets are projected onto a known regulatory network, seeking disease-associated subgraphs and specifically “hubs.” After giving the nodes a weight that reflects the confidence of the adjacent edges, the contribution to the weight of the hub of the enriched set is compared to chance. If the contribution is significant, the hub is proposed as key to the disease (Shu et al., 2016).

While the examples described so far focus on the identification of genes of interest, iOmicsPASS uses networks to discover signatures that distinguish between subgroups of samples and which, at the same time, form a graph of functional nodes, which can be used to learn about the associated phenotype. This implementation focuses on integrating transcriptome and proteome, normalizing gene expression with the number of copies. Moving on validated transcriptional regulation and PPI networks, the abundance of a pair of connected nodes becomes the weight of the edge that connects them. If the protein of a TF is elevated at the same time as its target, it is considered that the interaction is probable and it is given greater weight. Then, a nearest centroid algorithm looks for subgraphs that predict the phenotype, which constitute the classification signatures. The signatures obtained for breast cancer do not completely separate the Her2-enriched subtype from the luminal tumors, but divide the Her2+ group according to a subgraph linked to DNA replication and repair. Other interesting subgraphs are the enrichment of signalling by estrogen receptor and the regulation by FOXA1 and AP1 in the luminal B subtype compared to the luminal A, which in turn exhibits sub-expression of pathways related to the cell cycle. The basal subtype is not clearly defined when analyzing the omics separately, but it is when integrated, highlighting the importance of transcriptional regulation (Koh et al., 2019).

Following the idea of adapting a priori networks to the studied phenotypes, Glass et al. propose a tool that combines the three approaches to network integration. PANDA (*Passing Attributes between Networks for Data Assimilation*) starts from a probabilistic network and two a priori networks and finds a network that incorporates both sources, through the exchange of edges (Glass et al., 2013). PANDA is not a multi-omic integration tool, but rather a transcriptional network inference method based on the multi-layer formalism. However, algorithms have been built on PANDA to incorporate miRNA data, chromatin accessibility and omics in general, all through the fusion of probabilistic and a priori networks to the transcriptional network.

The reader interested in a comparative view of a priori network integration methods will found further information in Supplementary Table S6.

### 4.3 Multilayer networks

Multi-layer network integration consists of collapsing the different network layers into a single graph that adds the different sources of information. The layers are the networks derived from each omics under analysis, with the peculiarity of always including the same group of nodes. This allows to weigh the edges by the level of evidence and give strong support to any possible discovery. On the other hand, the restriction in terms of nodes requires an amount of information not always available. Reviewing the algorithm for single omics PANDA, it is possible to see these advantages and disadvantages.

PANDA seeks concordance between layers, refining each network with the information from the other, highlighting the aspects of the data that concern the a priori networks and the aspects of the a priori networks that best reflect the data. To this end, PANDA starts from: 1) a network inferred from the data, which is usually given by a correlation measure and provides the initial probability of co-regulation; 2) a known prior regulation network, consisting exclusively of nodes included in the data; and 3) a network of transcriptional factor interactions, with the same restrictions as the regulation network. In general, the idea is to combine the networks iteratively, each time with small changes governed by an update parameter and calculating a concordance score, whose convergence gives the stop signal. Concordance considers the accumulated evidence that gene j is regulated by TF i, given the cooperation (interaction) between i and other TFs that regulate j, and the co-expression of j with other genes regulated by i. Initially, the weight of all the edges is normalized to z-scores and is updated with the information shared between networks in each iteration, to end with a value that reflects the confidence in the interaction, being negative when the evidence says that there is no connection between the nodes and positive in the opposite case (Glass et al., 2013).

As can be seen from the PANDA premises, the algorithm is quite general and could be used to search for other transcription regulators, as is indeed the case. PUMA (PANDA Using MicroRNA Associations) is an adaptation that finds miRNAs and TFs, following the same steps, but starting with a transcript-miRNA co-expression network, the PPI network, a regulatory network that includes validated links with miRNAs and a list of regulators (miRNAs) that do not cooperate with each other and which should therefore not appear in the PPI network (Kuijjer et al., 2020). Although cooperation between regulators other than TFs has not been ruled out, there are also no established networks that can join the PPI network. Currently, as many expression predictors as desired could be integrated with PUMA, simply by adding them to the list of non-cooperators; alternatively, SPIDER allows considering the chromatin accessibility of a phenotype. The peculiarity of SPIDER (Seeding PANDA Interactions to Derive Epigenetic Regulation) is that the regulation network that feeds PANDA incorporates DNAse-seq data, so that the edges involve a TF motif that overlaps with an open chromatin region and with the regulatory region of the target gene, including sites outside the proximal promoter. Adding the information on chromatin accessibility predicts networks similar to those observed with ChIP-seq and works even better when the focus is on distal regulatory regions (Sonawane et al., 2021).

Thus, the so-called message passing allows for the discovery of new, phenotype-specific interactions that could not be found with prior network-guided integration and would be difficult to identify among the edges of probabilistic networks. However, in the case of PANDA, the accessible new interactions are limited to the mechanism where a regulator acts on co-expressed targets or cooperates with other regulators of the same target (Glass et al., 2013). Other tools that implement message passing are not subject to this restriction, because they are dedicated to classification.

SNF (*Similarity Network Fusion*) is an example of *message passing* for patient clustering. It starts by building similarity networks between samples according to the different omics, which are then combined iteratively, until the convergence of all the graphs into one. The final network incorporates both common and complementary information and contains a reduced amount of noise, by discarding the similarities without much weight. The integration with SNF of DNA methylation, transcript expression, and miRNA data from glioblastoma multiforme shows that each omics produces very different topologies, but together they sustain a subset of subtypes with significant differences in terms of survival. In the integrated network, 49.5% of the edges come from two omics and 17.2% from the three studied, the rest are only supported by one omic, but they represent great similarities. In addition to the classification as such, the networks help to understand the heterogeneity between patients, for example, the intra-subtype similarity can be traced to particular alterations, such as the overexpression of CTSD in the second group, which affects the response to the drug normally used to treat this type of cancer (Wang et al., 2014).

MONET (*Multi Omic clustering by Non-Exhaustive Types*) follows a similar strategy, starting with an omics similarity network and then searching for heavy and recurrent subgraphs between the omics, with a greedy algorithm. Each subgraph represents a module of similar patients, without necessarily having a connection between the modules or two modules arising from the same omics. This makes it possible to integrate incomplete omics and to identify atypical patients that do not fit into any module. The evaluation of the tool with methylation, transcript expression and miRNA expression of different types of cancer, yields a majority of modules based on a single omics and consistent groupings between restarts (Rappoport et al., 2020).

SNF and MONET share up to a certain point the omics similarity networks, making them suitable for few sample analysis and immune to the differences of scale and size between platforms. Although they differ in terms of criteria for integrating the networks of each omics, both tools are discarding the edges with less weight, giving them robustness against noise and heterogeneity of the data (Wang et al., 2014). In short, the properties of the message passing are desirable for cancer classification. If the goal is to search for multi-omics mechanisms, the message passing is also functional, as demonstrated by PANDA, PUMA and SPIDER, although there are alternatives.

The approach by Kim et al. starts off the same as SNF and MONET with omics similarity networks, in addition to a priori known omics interactions networks. Known regulatory pairs are integrated when expression of transcripts and miRNAs are incorporated; loci affected by CNVs are also considered, when this omics is included. Then, instead of iteratively fusing the networks, the coefficients are adjusted to obtain a linear combination of them, which appears to be the analytical equivalent of the previous examples. To find the best option, a study comparing the results of each tool is needed, as well as the properties of the algorithms, which, to the best of our knowledge, is currently unavailable. What can be said at the moment is that SNF/MONET would have an advantage, depending exclusively on omics, without needing prior networks. As has been observed previously, the results of Kim et al. based on more comprehensive information surpass those that depend on partial information. Coincidentally, there are isolated components in the integrated network, but most of the nodes are connected, suggesting that the different functional levels interact, even more, the authors suspect synergy between methylation and miRNAs in the regulation of expression (Kim et al., 2014).

Another example without *message passing*, just to integrate transcripts and proteins, starts from two independent co-expression networks, whose adjacency matrices allow computing the overlap between networks and finding conserved modules in both levels. Afterwards, the edges are filtered according to their association with the phenotype. The functional enrichment of the modules highlights the coordination between levels (Gibbs et al., 2014). In this case, the requirement of multilayer networks for the mapping between levels is very clear, although the relationship between transcripts and proteins is not 1:1, nor would it be between transcripts and methylation or miRNAs, exhibiting another advantage of PANDA and its variants.

In conclusion, networks allow for multi-omic integration with few compatibility issues across platforms. It is possible to distinguish, with some exceptions, three different approaches to network integration, each with its advantages and disadvantages, but clearly geared towards specific objectives. *Integration with probabilistic networks* is more versatile and allows for general observations on how different functional levels are connected, but the discussed articles have a clear bias towards the search for potential regulators and eventually towards the proposal of multi-omic mechanisms. A priori *networks* facilitate the finding of nodes of interest, with the expectation that predictors that exploit multiple types of data will be more robust and reflect the complexity of the disease (Gibbs et al., 2014). Finally, the *approaches that merge networks* pose a more complete classification of tumors, without necessarily assuming a common structure at all functional levels (Rappoport et al., 2020). The great exception are the PANDA derivatives, closer to a network *fusion* (merging) tool than to the rest of the approaches, but oriented towards the proposal of regulators.

To simplify the recovery and comparison of the networks produced by these tools, dedicated databases have been created. iNetModels contains multi-omic networks related to different metabolic conditions (Arif et al., 2021); while GRAND (Gene Regulatory Network Database) contains transcriptional and transcript-miRNA integration networks, obtained with PANDA derivatives, from data in the Cancer Cell Line Encyclopedia (CCLE) and TCGA (Ben Guebila et al., 2022). The ultimate purpose of these repositories is to facilitate the reuse of already published multi-omic networks and foster directed experimentation. However, even without the difficulties of distinguishing direct and indirect interactions or assigning direction to edges, it is necessary to remember that correlation is not enough to infer causality (Mousavian et al., 2016). Case uses, pros and cons of these approaches are presented in Supplementary Table S7.

### 4.4 New perspectives to face biomedical and clinical challenges using multiomic approaches

After giving a panoramic view of the methods, approaches and tools of multiomic integration, it may result helpful to consider how have these resulted useful to cope with challenging problems in the biomedical and clinical setting (Heo et al., 2021; Yoo et al., 2018). Here, we will discuss just a few examples of the many available, trying to present a glimpse of what lies ahead and what can be done.

One relevant field of application of multiomics and multiomic integration in cancer is the identification of novel biomarkers (Ivanisevic and Sewduth, 2023). In this regard, we can comment on the work by Liu and collaborators (Liu et al., 2021) that integrated multiomic data for 293 primary gastric patients from the TCGA collaboration analyzing copy number alteration and somatic mutation data to discover dysregulated factors in transcriptional regulation. These lead them to the identification of 31 molecular markers of genomic variation, including WASHC5 whose role could not be revealed using single omic methods. Using multiomic integration of genomic and transcriptomic data, Rahman and coworkers (Rahman et al., 2020) found evidence of GDF10 as a novel therapeutic biomarker for breast cancer, finding that downregulation of GDF10 is associated with breast cancer progression and patient’s survival, thus becoming a therapeutic biomarker for treating breast tumors. By integrating confederated data on mutations, copy number changes, expression changes, and protein–protein interactions from 12 different studies Li et al. (2020) were able to identify key biomarkers in ovarian cancer.

Multi-omic studies can also be useful to reveal new tumor types and subtypes (Gómez-Cebrián et al., 2021; Olivier et al., 2019). That was the case of the work by Yang and collaborators who developed a robust approach to identify clusters corresponding to cancer subtypes (Yang et al., 2022). Several applications of similar approaches can be found in connection to breast cancer (Nguyen et al., 2020; Wang Z.-z. et al., 2023; Sivadas et al., 2022), glioblastoma (Yuan et al., 2020; Park et al., 2019), hepatocellular carcinoma (Ouyang et al., 2020), lung adenocarcinoma (Zhao et al., 2021; Wang et al., 2022), and ovarian cancer (Guo et al., 2020).

Therapeutic designs can also benefif form multiomic analyses (Celebi et al., 2019; Zielinski et al., 2021). There are examples of the usefulness of integrating omic analytics in the design of novel drug combinations to treat drug-resistant multiple myeloma (Kumar et al., 2022), to identify novel drug targets for clear cell renal carcinoma immunotherapy (Reustle et al., 2020), to find new therapeutic targets for esophageal squamous cell carcinoma (Jin et al., 2021) and even to tackle with muyltiple tumor types using a pancancer approach (Ali et al., 2018).

These are but a handful of the real world applications of multiomic integrative analytics, however we hope that these may help the readers to look up into other applications of these extremely versatile sets of tools and methods.

## 5 Conclusion

Along this review article we have discussed some issues on multi-omic data integration that we considered fundamental when considering studies on cancer biology. These issues are by necessity incomplete and biased to our own research experience. However, we consider that these may serve as a good starting point for scientists investigating the exciting cues that current omic technologies may provide on our integrated understanding of molecular oncology.

Among these issues we can mention the following (stated in the form of research highlights):

• We have introduced concept of multi-omic integration, which combines data from various omics layers to gain a comprehensive understanding of biological systems. Emphasizing the importance of integrating different types of omics data to uncover complex interactions and mechanisms underlying biological processes and diseases, while reviewing some challenges and opportunities in multi-omic integration, including data heterogeneity, scalability, and interpretation of integrated results.

• We have introduced the concept of single omic networks, representing interactions within individual omics layers, such as gene regulatory networks derived from gene expression data. While recognizing that the network approach is just one of several integration alternatives, we discussed various methods for constructing single-omic networks, including correlation-based approaches, mutual information, and machine learning algorithms, and provide examples of applications of single-omic networks, such as identifying key regulatory genes and pathways associated with diseases and biological processes.

• Given the high variability of the underlying biological phenomena involved in cancer settings and the intrinsic and extrinsic sources of experimental noise, we have also stress the relevance of probabilistic modeling approaches. We mentioned how probabilistic networks are able to capture uncertainty in biological interactions and enable the integration of multiple sources of evidence. Introduced Bayesian networks and Markov random fields as examples of probabilistic network models used in bioinformatics and discussed the advantages of probabilistic networks, such as their ability to handle noisy data, incorporate prior knowledge, and provide probabilistic inference of biological relationships.

• Full integration implies also considering prior knowledge available. In this regard, a priori networks, depicting existing knowledge of biological interactions and pathways obtained from literature, databases, and experiments were considered. Examining how a priori networks can serve as valuable prior information for guiding the integration of multi-omic data and interpreting integrated results.

• The relatively nascent field of multilayer networks is introduce as a novel paradigm to fully integrate multiomic data into a single (somewhat coherent) framework. We briefly considered algorithms and tools for multilayer network integration, such as PANDA, PUMA, SPIDER, SNF, and MONET, which leverage message passing and iterative optimization to uncover phenotype-specific interactions and classify tumors.

In brief, we have presented a panoramic view of some contemporary approaches for integrating multi-omic data, including probabilistic networks, a priori networks, and network fusion methods, each with its advantages and applications in understanding the complex biological phenomena related to tumor biology in the context of biomedical research.

The future of data and network integration in the multi-omics and cancer research, presents thus vast opportunities for advancing our understanding of complex biological systems. As omics technologies continue to generate increasingly large and diverse datasets, the integration of these datasets through sophisticated computational and statistical methods will be essential for extracting meaningful insights. The development of more robust and scalable integration methods, such as those that can handle data heterogeneity, noise, and the high dimensionality typical of multi-omics data, will be crucial. These methods must be capable of discerning subtle biological signals from background noise and integrating prior knowledge to make accurate predictions about disease mechanisms and therapeutic targets.

Additionally, the integration of network-based approaches offers exciting prospects for uncovering the intricate web of interactions that underlie biological processes. The ability to construct and analyze multilayer networks that incorporate data from different omic layers—such as genomics, transcriptomics, and proteomics—will enable researchers to capture the complexity of molecular interactions and their impact on phenotypes. Future developments in this area may include the refinement of algorithms that can efficiently fuse multiple omic datasets into cohesive networks, as well as the application of machine learning techniques to identify key regulatory nodes and pathways. These advancements could lead to more personalized and effective treatments by revealing novel biomarkers and therapeutic targets specific to individual patients or subgroups, thus pushing the boundaries of precision medicine.

As we look forward, the integration of increasingly complex and high-dimensional data, alongside the development of more sophisticated network models, will likely play a pivotal role in the next- generation of biomedical research and healthcare.
